# Mental stress in the workers exposed to humidity in a cheese processing factory

**DOI:** 10.4103/0019-5278.40815

**Published:** 2008-04

**Authors:** SM Shushtarian, AH Hajipour, Y Rastegari

**Affiliations:** Tehran Medical Branch, Islamic Azad University, Tehran, Iran; * Cultural Handicraft and Tourism, Higher Education Centre, Tehran-Iran

**Keywords:** cheese processing factory, humidity, mental stress

## Abstract

Certain inevitable physical factors in working environment can damage the workers in related fields. Sea sickness and white finger due to ship movement and vibration respectively are two examples in this regard. Humidity in working area can also bring discomfort of the workers in humid area. Cheese processing factories are such places where there is high humidity in the working space.

Mental stress is a psychological complication which can arise due to some physical factors in certain occupational activities, therefore an attempt was made to have a search on mental stress in the laborers working in a cheese factory in Orumieheh, a city in north of Iran, with a cold climate throughout the year.

For the purpose of the present study, a cheese processing factory with 100 workers was selected. The workers were divided in to two groups. One group was exposed to high humidity and the other exposed to normal humidity level. A standard questionnaire was given to two groups to check the mental stress.

The results obtained from both groups were compared.. The result showed severe mental stress in workers exposed to high humidity whereas moderate stress level in other workers.

The conclusion of the present work is a proof of the adverse effect of humidity in working environments which reflect in mental stress in workers which will be discussed in detail in full paper.

## INTRODUCTION

Psychological complications are an inseparable part of industrial life..[1–3] Screening of these complications is important as far as social health is concerned. Part of these complications are due to physical factors existing in different occupational activities.[4–5]

Heat and humidity in certain occupational activities are factors that can bring about discomfort to laborers working in these areas.[6–10] It is already reported that the temperature ranges (23 ± 2)°C and humidity ranges (50 ± 10)% are suitable for the workers in hot and humid environments.[11] Therefore exceeding the above mentioned ranges would bring about discomfort of the laborers.

Accidentally, it was observed that some of the laborers working in a cheese processing factory were suffering from mental stress. On this basis, a research was planned out in one of the cheese processing factory with high humidity and relatively cold temperature, to look for the possibility of mental stress in laborers working in the factory.

It is beneficial to mention that there are no references available on the effect of the only humidity on psychological complications and that too mental stress in cheese processing factories where as there are large number of such factories in developed countries. This may indicate the importance of such research in the concerned area.

## MATERIALS AND METHODS

A cheese processing factory in the North of Iran, Orumieheh was selected to be studied in the winter season when the climate is cold. Percentage humidity (% HR) and temperature (°C) were recorded in different parts of the factory. The selection of the places where the measurements were done was according to site, the laborers used to work in the factory. Hygrometer, HD 100 was the instrument used for the purpose of present work.[12]

To test the mental behavior of the laborers, the factory was classified in two sections. The first section was the places in the factory where the laborers encountered with suitable humidity like the offices, laboratory and spaces outside the factory. The laborers in these areas were taken as control group. The second area was a place where high humidity above 60% was persistent. The laborers in these areas were taken as case group. One hundred laborers (60 and 40 as case and control group respectively) were selected from two sections. Selection was done in way that the laborers match in different aspects except humidity levels which was different. Questionnaire Lionel Coudron[13] was used to compare the psychological behavior i.e. mental stress in two groups.

The results obtained in two groups was compared and searched for the possible differences in two groups as far as the mental stress was concerned.

## RESULTS

Certain factors in working areas can bring about psychological disturbances. The result of present research is a clue for this comment.

Table 1, is the measurement of humidity per cent and temperature (°C) in different locations of a cheese processing factory respectively. As it is observed in table 1, the temperature measurements in the administrative section i.e. in the office lies in normal range whereas in all other places the temperature is below the standard range and only in one place means the place where the boiling of milk takes place is above the standard range i.e. 35°C.[11]

**Table 1:** Temperature (°C) & % humidity measurements in different areas of a cheese processing factory.

Location No.	Location	% Humidity	Temperature °C
1	freezing room (below 0 °C)	43.5 ± 1	-10 ± 2
2	working office	46.0 ± 2	21 ± 2
3	general laboratory	46.6 ± 2	11 ± 4
4	packing machine	47.7 ± 2	15 ± 2
5	Window	47.8 ± 1	21 ± 1
6	freezing room (above 0 °C)	52.7 ± 2	7 ± 1
7	humidity maintaining machine	54.9 ± 2	18 ± 2
8	laboratory (microbial)	57.4 ± 3	14 ± 2
9	outside factory	57.6 ± 3	3 ± 2
10	stretching machine	58.8 ± 2	17 ± 1
11	milk reservoir	59.9 ± 3	5 ± 3
12	milk boiling pot (pasteurizing instrument)	65.0 ± 3	19 ± 3
13	pasteurizing instrument (milk balance)	68.0 ± 3	19 ± 3
14	cheese testing room	69.0 ± 3	17 ± 2
15	fermentation machine (wet)	71.0 ± 3	17 ± 1
16	boiling pot	78.4 ± 2	35 ± 2

As the humidity per cent is concerned, we can divide the factory in two parts. In one part the humidity lies in normal range means between 40-60% whereas in other parts where the milk processing activity is performed the humidity is above 60%.

**Graph 1:**
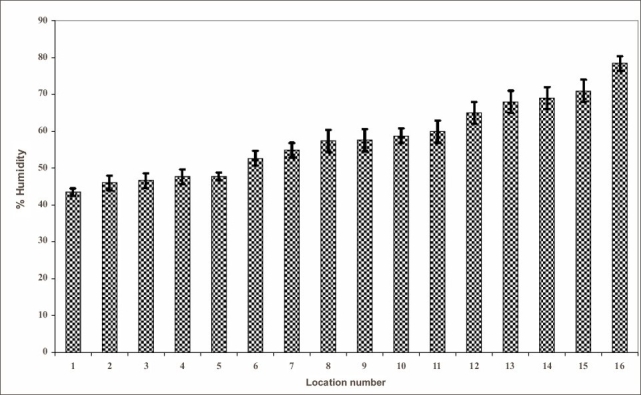
Percentage of humidity in different locations of a cheese processing factory

**Graph 2:**
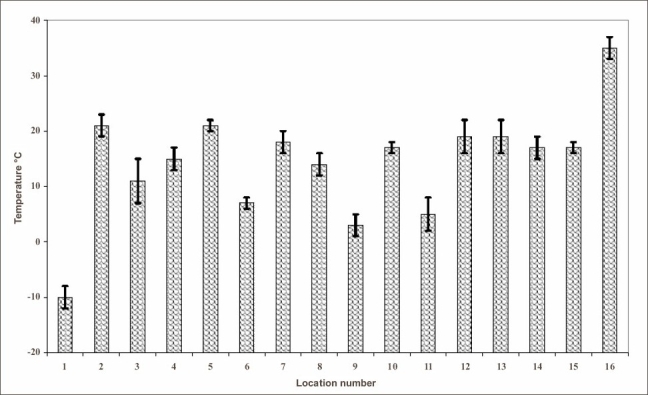
Temperature (°C) in different locations of a cheese processing factory

Graph 1 and 2, are the graphical presentation of Table 1 and 2.

**Table 2:** Mental stress levels measurement of the labors (%) working in different humidity levels

Humidity\Stress levels (%)	Natural < 30	Moderate (30-60)	Severe < 90
Suitable	12.5	80.0	7.5
Severe	3.3	8.3	88.30

Table 2 shows the result obtained from Lionel Coudron questionnaires.[13] As it is observed from the table 2 the laborers working in normal range of humidity but in a cold place suffer from moderate stress level, i.e. rank between 30 to 60 where as the ranking exceed 60 in laborers working in humid places of the cheese processing factory and finally very few laborers liein the natural range of stress level. It may be useful to mention that the laborers had suitable clothing to protect them from cold.

**Graph 3:**
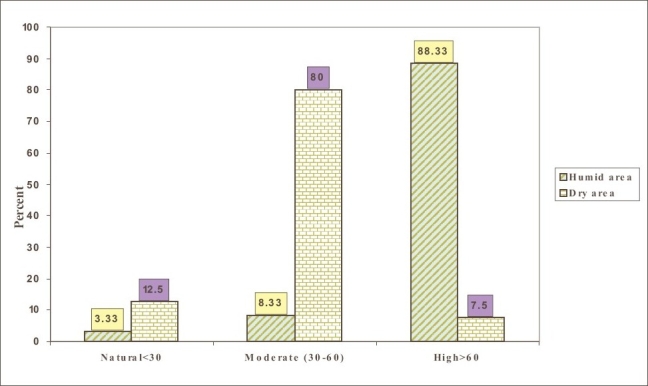
Stress levels in labors (%) working in a cheese processing factory

Finally Graph 3, is the graphical presentation of Table 2

## CONCLUSION

The aim of present work is to show the effect of humidity on psychological behavior of the workers working in a cheese processing factory. There are large number references available on heat stress whereas there are no references available on effect of the only humidity on mental stress.

The present research contains two main characteristics. The first is humidity, which is taken into consideration for the purpose of present study and the second is the cheese processing factory..No references are available in this connection.

As it is obvious in table 1, the temperature lies below the suitable range whereas the humidity is high than the recommended and suitable range. The problem of cold environment is solved by suitable covering for the laborers.

The workers under study were tested for mental stress and the result obtained from table 2 shows that, the laborers exposed to standard humidity have moderate mental stress whereas the laborers working in higher humidity level suffers from severe mental stress. The difference between these two groups was statistically significant (p < 0.005).

Therefore the conclusion of present work is the effect of humidity on workers of a cheese processing factory which is nothing but mental stress.
